# Intensity and frequency of moral distress in mental health nurses in Brazil*

**DOI:** 10.1590/1980-220X-REEUSP-2023-0122en

**Published:** 2023-09-04

**Authors:** Mario Sergio Bruggmann, Dulcinéia Ghizoni Schneider, Flávia Regina Souza Ramos, Graziele de Lima Dalmolin, Jeferson Rodrigues, Ácmon Bhering

**Affiliations:** 1 Secretaria de Estado da Saúde de Santa Catarina, Florianópolis, SC, Brazil.; 2 Universidade Federal de Santa Catarina, Programa de Pós-Graduação em Enfermagem, Florianópolis, SC, Brazil.; 3 Universidade Federal de Santa Maria, Departamento de Enfermagem, Santa Maria, RS, Brazil.; 4 Universidade Federal de Santa Catarina, Departamento de Enfermagem, Florianópolis, SC, Brazil.; 5 Secretaria Municipal de Educação de Florianópolis, SC, Brazil.

**Keywords:** Psychological Distress, Mental Health, Ethics, Nursing, Working Conditions, Distrés Psicológico, Salud Mental, Ética en Enfermería, Condiciones de Trabajo, Psychological Distress, Mental Health, Ethics, Nursing, Working Conditions

## Abstract

**Objective:**

To assess the intensity and frequency of moral distress in mental health nurses in Brazil.

**Method:**

Cross-sectional study with 173 nurses from the Psychosocial Care Network in Brazil. The Brazilian Scale of Moral Distress in Nurses, adapted for the context of mental health, was used. For data processing, descriptive and inferential statistical analysis was used.

**Results:**

Mostly moderate levels of intensity and frequency of moral distress (medians between 2.25 – 3.73 and 2.00 – 3.22, respectively) were observed, with emphasis on the factors working conditions and social conflicts.

**Conclusion:**

The level of moral distress evidenced in mental health nurses in Brazil reflects the dimension and amplitude of the phenomenon in different points of the Psychosocial Care Network. The relevance of discussions on coping strategies for moral distress is highlighted, articulating elements such as sensitivity, resilience, and moral courage, so that ethical deliberation is applied in care and management settings.

## RESUMEN

**Objetivo:**

Evaluar la intensidad y frecuencia del sufrimiento moral en enfermeros de salud mental en Brasil.

**Método:**

Estudio transversal con 173 enfermeros de la Red de Atención Psicosocial del territorio brasileño. Se utilizó la Escala Brasileña de Sufrimiento Moral en Enfermeros adaptada para el contexto de la salud mental. Se utilizó análisis estadístico descriptivo e inferencial para el procesamiento de datos.

**Resultados:**

Se observó, en su mayoría, niveles moderados de intensidad y frecuencia de sufrimiento moral (medianas entre 2,25 – 3,73 y 2,00 – 3,22, respectivamente), con énfasis en los factores condiciones de trabajo y conflictos sociales.

**Conclusión:**

El nivel de sufrimiento moral evidenciado en enfermeros de salud mental en Brasil refleja la dimensión y amplitud del fenómeno en los diferentes puntos de la Red de Atención Psicosocial. Se destaca la relevancia de las discusiones sobre estrategias de enfrentamiento del sufrimiento moral, articulando elementos como la sensibilidad, la resiliencia y el coraje moral, para que la deliberación ética sea aplicada en contextos de cuidado y gestión.

## INTRODUCTION

Moral distress is defined as an emotional imbalance manifested when nurses are exposed to a conflicting situation and identify the morally correct action to be taken, but structural and/or relational barriers prevent them from acting according to their values^(1)^. Its occurrence is associated with the subjective conditions and experiences of the nurses’ work process, which require ethical positions, clinical decisions, care and conflict management, expressed through manifestations of frustration, anger, physical and emotional exhaustion, and professional impotence^(2,3)^.

From a procedural perspective, moral distress is considered an ethical/moral experience manifested in the face of a moral problem (starting point), requiring some degree of sensitivity, concern, and moral discomfort by the nurse, so that their moral judgment occurs in a more prudent way^(4)^. In care practice, failures in this process lead to changes and personal and professional resonances in nurses, and bring out negative impacts to the people they assist^(2)^.

In the field of mental health, materialized in the Brazilian scenario by the different points of the Psychosocial Care Network (*RAPS*), the nurse acts directly in the management of services and assistance for people with mental disorders and for those with needs arising from the use of alcohol and other drugs, supported by Resolution No. 678/2021, which regulates the performance of the nursing team in this field of work^(5)^.

For this context, it is recommended that nurses develop ethical and technical skills for a humanized, comprehensive, and community-based work, based on the users’ needs^(6)^. However, contrary to this perspective, problems are found related to professional training, lack of understanding of the policies and care model in force in mental health care and, above all, to the very disarticulation of *RAPS*, which contributes to the discontinuity of care^(7)^.

These particularities, added to the different ethical/moral problems and disrespect for users’ rights, work overload, staff deficit, inflexible organizational cultures, insufficient professional recognition, and low remuneration, are predictors of moral distress in mental health nurses^(8,9)^.

In view of this panorama, for professionals to develop effective skills for coping with ethical/moral and institutional problems in response to moral distress, their generating situations shall be clearly defined. There is a certain limitation of Brazilian studies covering the theme in mental health nurses, and only an integrative literature review^(8)^ and a qualitative study^(9)^ were identified. Studies on moral distress in this specific population predominate in the international scenario, where countries such as Japan^(10)^, South Korea^(11)^, Jordan^(12)^, U.S.A^(13)^, Italy^(14)^, Norway^(15)^, Thailand^(16)^, and Iran^(17)^ devoted more attention to this issue.

Based on the issue raised and considering the particularity of Brazil, a continental, multicultural country with significantly different sociodemographic aspects, as well as the absence of observational studies on the dimension of moral distress in nurses working in *RAPS*, the relevance of investigating the object is warranted. Therefore, the present study aimed to evaluate the intensity and frequency of moral distress in mental health nurses in Brazil.

## METHOD

### Design of Study

Cross-sectional study with non-probability, ­convenience sampling^(18)^.

### Local and Population

The study was carried out in the Brazilian *RAPS*, distributed in the 27 units of the federation (26 states and the Federal District). Nurses working in Psychosocial Care Centers (*CAPS*) (type I, II and III, CAPSi - childhood and adolescence, CAPSad and CAPSad III - alcohol and drug users), specialized psychiatric (public) hospitals, specialized reference units in general hospitals, mental health clinics, and street clinics were included, regardless of the time since the end of the undergraduate course, time of practice, or type of employment. To calculate the estimated N of the population (12,294), the number of registered services mentioned above and their geographic distribution, Resolution No. 543/2017 of the Federal Nursing Council (which provides for the dimensioning of nursing staff) and the Technical Note No. 11/2019 (establishes the minimum composition of nurses in mental health services) were considered.

### Sample Definition

The sample size was calculated using the finite population formula for epidemiological studies, with a confidence level of 95% and sampling error of 10% of the population average. With this calculation, the resulting minimum sample was 85 participants. However, 173 nurses participated in the study, with regional representativeness (quotas), in a similar distribution to the general population calculated for the study. It should be noted that the increase in the sample decreased the sampling error to 7%, considering the same confidence standards.

### Data Collection

Data were collected online, via google.docs, from March to June 2021, through the Brazilian Scale of Moral Distress in Nurses adapted for the context of mental health (EDME-Br-SM). This measurement instrument was adapted and validated through a methodological study^(19)^, based on the Brazilian Scale of Moral Distress in Nurses^(2)^.

The process considered the criteria, content and construct validation steps, in eight moments: 1) determination of what to be measured (moral distress in mental health nurses^(8)^); 2) generation of a set of items – criterion validation (analysis and comparison of items from the original instrument, considering adjustments, inclusions, and exclusions); 3) determination of the measurement format (two Likert scales to measure moral distress for intensity, ranging from 0 (none) to 6 (for very intense suffering) and frequency, ranging from 0 (never) to 6 (very frequent); 4) review by experts - content validity (items evaluated by 14 experts in moral distress and 20 in mental health, according to their experiential and cultural context, in terms of relevance, clarity, and consistency. Pre-test with 30 mental health nurses and application of the Content Validity Index (CVI) 5) inclusion of items (predictors were reviewed); 6) application of the instrument in a sample of interest (173 mental health nurses from different Brazilian regions); 7) item evaluation – criterion validity (statistical analysis); 8) scale optimization (instrument validated with 37 items and seven factors).

The EDME-Br-SM includes the following sociodemographic variables: age, sex, region of the country, time since end of undergraduate course, complementary and post-graduate training, number of employment relationships, nature and type of employment, level of care in which they work, time of practice in the main job, weekly workload, and type of service/unit in which they work. Its predictors were organized into the following factors: Factor 1 (F1) – Working conditions, with nine items; Factor 2 (F2) – Defense of values and rights, with five items; Factor 3 (F3) – Safety and professional autonomy, with seven items; Factor 4 (F4) – Ethical violations, with four items; Factor 5 (F5) – Social conflicts, with five items; Factor 6 (F6) – Professional ethical competence, with three items; and, Factor 7 (F7) – Conflicts with Management, with four items.

To access the participants, social media, telephone contact with the *RAPS* devices, and messages through application were used. The snowball sampling technique was used, which is a non-probabilistic sampling technique, where the selected sample indicates new participants in their network of acquaintances, through linear sampling. To access the instrument, the respondent received a redirection link to the google.forms platform to access the Free Informed Consent Form signed by the authors, informing the nature and objectives of the study. After agreeing to the term, the participant should click on “I agree to participate in the study” to access the instrument itself.

### Data Analysis and Treatment

Data were stored and organized in spreadsheets in Microsoft Excel 2010 and exported for analysis through the software SPSS (Statistical Package for Social Sciences), version 25.0. For the purposes of statistical analysis of the scores of intensity and frequency of moral distress in the EDME-Br-SM, the following intervals were considered as parameters: low (0–1.99), moderate (2.00–3.99), and high (4.00–6.00)^(20)^.

Descriptive statistical analysis was used with relative and absolute frequency distribution for sociodemographic and work variables. The analysis of intensity and frequency of moral distress for each EDME-Br-SM factor was presented by medians and interquartile ranges (Q1-Q3). The Shapiro-Wilk test was performed to test data normality. Sequentially, non-parametric tests of Mann-Whitney were performed to compare the distributions of moral distress between men and women, and the Kruskal-Wallis test to compare the distributions of moral distress between the categories of other variables, with the null hypothesis being rejected when p < 0.05.

### Ethical Aspects

The study was approved by the Human Research Ethics Committee of the Universidade Federal de Santa Catarina, under Opinion 4.193.686, 2020, in compliance with Resolution Nº 466/2012 of the National Health Council and other complementary provisions that deal with guidelines and standards that regulate research involving human beings.

## RESULTS

In the sample of 173 nurses working in mental health services in Brazil, there was a prevalence of female participants (n = 137; 79.2%), aged between 30 and 39 years (n = 91; 52.6%), from the Southeast region (n = 61; 35.3%), with graduate certificate (n = 98; 56.7%), an employment relationship (n = 106; 61.3%), in the public sector (n = 142; 82.1%), permanent work bond (n = 83; 48%), in secondary level of health care (n = 92; 53.2%), working time in the main job for up to 5 years (n = 76; 43.9%), with a weekly workload of 31 to 40 hours (n = 82; 47, 4%), working in different CAPS modalities (n = 93; 69.5%). There was variability in the participants’ time after undergraduate course, and the results were similar in the periods of 0–5 years (n = 44; 25.4%), 6–10 years (n = 46; 26.6%), and 11–15 years (n = 43; 24.9%). The analysis of the medians and interquartile ranges (Q1-Q3) of intensity and frequency of the EDME-Br-SM factors is shown in Table 1, while the analysis of the instrument’s items is shown in Table 2.

**Table 1:** Intensity and frequency of moral distress in mental health nurses according to the EDME-Br-SM factors - Florianópolis, SC, Brazil, 2021. n = 173.

Variables	Median (Q1-Q3)	
	Intensity	Frequency
F1 – Working conditions	3.33 (1.88–5.44)	3.22 (1.78–5.22)
F2 – Defense of values and rights	1.60 (0.20–3.60)	1.40 (0.20–2.80)
F3 – Professional safety and autonomy	3.00 (1.57–5.29)	2.86 (1.57–4.86)
F4 – Ethical violations	2.50 (0.50–4.50)	2.00 (1.00–3.50)
F5 – Social conflicts	3.20 (1.60–5.20)	3.00 (1.60–4.80)
F6 – Professional ethical competence	3.00 (1.33–5.00)	3.00 (1.67–5.00)
F7 – Conflicts with management	2.25 (0.75–5.00)	2.00 (0.75–3.75)

**Table 2:** Intensity and frequency of moral distress in mental health nurses according to the EDME-Br-SM predictors - Florianópolis, SC, Brazil, 2021. n = 173.

Variables	Median (Q1-Q3)	
	Intensity	Frequency
**F1 - Working conditions**		
Recognize that permanent materials are insufficient	3.00 (2.00–5.00)	3.00 (2.00–5.00)
Recognize that consumables are insufficient	3.00 (2.00–6.00)	3.00 (2.00–5.00)
Recognize that consumables are inadequate	3.00 (2.00–5.00)	3.00 (2.00–5.00)
Recognize that permanent materials are inadequate	3.00 (1.00–5.00)	3.00 (1.00–5.00)
Recognize that the lack of permanent education support impairs the work process	4.00 (2.00–6.00)	4.00 (2.00–6.00)
Recognize that disarticulations in the Psychosocial Care Network impair access and care for users and/or family members	5.00 (2.00–6.00)	4.00 (2.00–6.00)
Work with an incomplete multidisciplinary health team	3.00 (2.00–5.00)	3.00 (2.00–5.00)
Work with an insufficient number of nursing professionals due to the service demands	3.00 (2.00–5.00)	3.00 (1.00–5.00)
Experience situations of work overload	3.00 (2.00–6.00)	3.00 (2.00–5.00)
**F2 – Defense of values and rights**		
Recognize situations of disrespect for the user’s right to confidentiality/secrecy	2.00 (0.00–3.00)	1.00 (0.00–3.00)
Recognize situations of disrespect for the right of users and family members to information	1.00 (0.00–3.00)	1.00 (0.00–2.00)
Recognize situations of disrespect for the user’s right to privacy/intimacy	2.00 (0.00–4.00)	2.00 (0.00–3.00)
Recognize situations of mistreatment by professionals in relation to the user	1.00 (0.00–4.00)	1.00 (0.00–3.00)
Experience professional behaviors that disregard users’ beliefs and culture	2.00 (1.00–4.00)	2.00 (1.00–3.00)
**F3 – Professional safety and autonomy**		
Feel undervalued compared to other professionals	3.00 (1.00–5.00)	3.00 (1.00–5.00)
Perform actions that are not inherent to the role	3.00 (1.00–5.00)	3.00 (1.00–5.00)
Recognize that the physical structure of the service is inadequate	3.00 (2.00–6.00)	3.00 (2.00–5.00)
Experience routines and practices that are inappropriate for professional safety	3.00 (2.00–5.00)	3.00 (2.00–5.00)
Recognize that the physical structure of the service is insufficient	3.00 (2.00–5.00)	3.00 (2.00–5.00)
Experience conflicts related to the attributions of members of the multidisciplinary team	3.00 (2.00–5.00)	3.00 (2.00–5.00)
Have limited professional autonomy in decision-making processes	3.00 (1.00–5.00)	2.00 (1.00–4.00)
**F4 - Ethical violations**		
Recognize acts of negligence by nurses	2.00 (0.00–4.00)	2.00 (1.00–3.00)
Recognize acts of recklessness by nurses	2.00 (0.00–4.00)	2.00 (1.00–3.00)
Recognize acts of recklessness by other team professionals	3.00 (1.00–5.00)	2.00 (1.00–4.00)
Recognize acts of recklessness by other team professionals	3.00 (1.00–5.00)	2.00 (1.00–4.00)
**F5 - Social conflicts**		
Feel pressured by the user/family member in a situation they cannot intervene in	2.00 (1.00–4.00)	2.00 (1.00–4.00)
Feel powerless to defend the user/family member in situations of social vulnerability	3.00 (1.00–6.00)	3.00 (1.00–5.00)
Recognize that situations of family abandonment negatively interfere with the adherence and resolution of the user’s treatment	5.00 (3.00–6.00)	5.00 (3.00–6.00)
Recognize that ineffective communication between members of the multidisciplinary team leads to harm to care	3.00 (2.00–6.00)	3.00 (2.00–5.00)
Experience situations of physical and/or verbal aggression by the user/family member in relation to professionals	3.00 (1.00–4.00)	2.00 (1.00–4.00)
**F6 - Professional ethical competence**		
Work with nursing assistants/technicians with an inadequate profile and/or technical preparation for the area	3.00 (1.00–5.00)	3.00 (2.00–5.00)
Work with nurses with an inadequate profile and/or technical preparation for the area	3.00 (1.00–5.00)	3.00 (1.00–5.00)
Work with professionals from other categories with an inadequate profile and/or technical preparation for the area	3.00 (2.00–5.00)	3.00 (2.00–5.00)
**F7 - Conflicts with management**		
Feel disrespected by service managers	2.00 (1.00–5.00)	2.00 (1.00–4.00)
Recognize ethically inadequate attitudes from managers	3.00 (1.00–5.00)	2.00 (1.00–4.00)
Feel pressured to compromise or remain silent in the face of irregularities committed for the benefit of the institution	2.00 (0.00–5.00)	2.00 (0.00–3.00)
Feel impotent to defend the user’s autonomy in care decisions	2.00 (1.00–5.00)	2.00 (1.00–4.00)

According to the statistical tests used, no association was found between the variables sex, region, time since end of undergraduate course, education, nature of the employment relationship, level of health care, and moral distress (p > 0.05). In the variable age, a higher median value for moral distress was observed in people between 40 and 49 in factor 7 “Conflicts with management” (p < 0.05). For the other factors, there were no statistically significant differences between the distributions of moral distress by age group.

There was a statistical difference in the frequency distributions of moral distress in the variable number of employment contracts in the factor “Professional ethical competence”, indicating greater moral distress in nurses who work in three or more jobs, compared to those who work less. The predictive situations of moral distress in this factor also had a greater association with nurses working in a *CLT* regime (Brazilian regime which gives no guarantee of permanence), compared to other work regimes. Conversely, nurses who work up to 30 hours a week had the highest values of moral distress in the predictors of the factor “Safety and professional autonomy” in relation to those who work more hours (p < 0.05).

The variable time of practice in the main job was associated with the factors “Safety and professional autonomy”, “Social conflicts” and “Conflicts with management”. The two groups with the greatest moral distress for these factors were nurses whose work practice time ranges from a period between 6 and 10 years and 11 and 15 years. There is also an association between the service in which the nurse works and moral distress. Nurses from CAPS III showed greater intensity of moral distress in the factor “Safety and professional autonomy”; those from CAPSi, in the factor “Ethical violations”; and those from the mental health outpatient clinics, in the factor “Defense of values and rights” (p < 0.05).

In Table 1, it is noted that only the factor “Defense of values and rights” presented a value considered low and such discrepancy indicates a possible heterogeneity of the phenomenon among nurses in this factor, pointing to a probable influence of sociodemographic and labor variables on moral distress. In the opposite direction, it is observed that the other factors presented moderate values for intensity and frequency of moral distress, with emphasis on “Work conditions” and “Social conflicts”.

In Table 2, the predictors “Recognize that the lack of permanent education support impairs the work process”, “Recognize that disarticulations in the Psychosocial Care Network impair access and care for the user and/or family member” and “Recognize that situations of family abandonment negatively interfere with adherence and resolution of the user’s treatment” showed the highest values for median and third quartile (75%). These predictors had the maximum score in the third quartile of moral distress, that is, at least 25% of the nurses who participated in the study assigned the maximum score for both the intensity and the frequency of these items.

## DISCUSSION

From the results of intensity and frequency of moral distress according to the scale factors, moderate to high levels of the phenomenon were mostly observed, with “Working Conditions” and “Defense of values and rights”, with the highest and lowest medians, respectively, being highlighted.

For the predictors related to the factor “Working conditions”, with moderate to high levels for the intensity and frequency of moral distress, it is considered that the effective articulation of the *RAPS* services promotes comprehensive assistance to users in the different network devices^(8)^. Thus, complete communication between professionals and services will creatively encourage other processes relevant to nurses’ work to take place, such as the collective construction of knowledge and the implementation of care technologies, based on permanent health education. However, for this scenario to be configured and to strengthen nurses in situations of moral distress in this factor, it is assumed that the correct dimensioning of nursing is a determining factor, thus reducing work overload and providing safe means for qualified professional care^(2)^.

The items of the factor “Defense of values and rights” refer to the user’s right to humanized, integral, private, and safe assistance, where their autonomy and values are respected. However, when these aspects are violated by a member of the multidisciplinary team, nurses can experience moral distress. In this sense, it is worth noting that disrespect for users’ autonomy is directly correlated to the phenomenon and affects mental health care nurses^(10,13,15,16)^. In the present study, the factor aforementioned had the lower median values for intensity and frequency of moral distress, the same result presented by a Brazilian study on moral distress in nurses from other work settings^(2)^. It is believed that these values are related to the expansion of discussions on ethical and technical competences in mental health, with emphasis on communication skills, respect for people with mental disorders and their families, guarantee of rights, autonomy of users in their treatment, and effectiveness of community-based services in this process^(7-9)^.

Nevertheless, even in the face of findings with lower values, the predictors of this factor still generate moral distress in mental health nurses and so, have to be discussed. Therefore, it is worth noting that the defense of users’ sociocultural rights and values is an advocacy practice carried out by nurses, which seeks to strengthen and qualify their autonomy^(21)^, providing guidance on ethical, technical assistance that is free from damage arising from malpractice, negligence, or imprudence.

From the perspective of the users’ rights defense, it is worth mentioning that opposing forces pointing to the professional non-compliance with their professional code of ethics are related to the factor “Ethical violations” and generate moral distress^(2)^. In this factor, the items related to recognizing acts of imprudence and negligence on the part of other professionals in the team had the highest values for moral distress among the block items. Besides rescuing differences related to training and professional skills in dissimilar scenarios, these predictors of moral distress also refer to the historical and social context of mental health care, closely associated with the curtailment of rights, neglect, abuse, disrespect, and abandonment of the person with mental disorder, either by the State, society, or professionals^(6,22)^.

The multifaceted ethical problems emerging from the practice of mental health care nurses are strongly associated with moral distress, causing these professionals to distance themselves from the profession^(10,12,13,15,23)^. However, respecting the law of professional practice and ethical principles constitutes the basis of acting ethically that can minimize situations leading to the phenomenon. In this regard, the construction of practice based on ethical, technical, and legal precepts of the profession is an element that contributes to the visibility and empowerment of nurses in different work contexts^(16,24)^ and is related to the factor “Safety and professional autonomy”.

To exercise their autonomy, nurses have to associate their technical-scientific knowledge with the attentive observance of their rights, duties, and prohibitions, considering the ethical education that marks their professional identity. In this direction, the elements “recognition, power and identity”^(2)^ relate to the factor “Safety and professional autonomy”, considering that autonomy and knowledge are associated and raise the level of nursing in the scientific and social scenario.

The factor “Social conflicts” is particularly linked to the context of mental health services in Brazil and presented the second highest median for the intensity and frequency of moral distress, with the highest predictor of the instrument “Recognizing that situations of family abandonment negatively interfere with the adherence and resolution of the user’s treatment” (approximately 25% of the sample assigned the maximum score for the intensity and frequency of the item). It is believed that the highlight of the predictor and the factor in general in the study is related to a complex Brazilian historical-social paradigm of inequality, which gives way to a significant number of people with low family income and limited access to health, education, and culture services, thus perpetuating a community that insufficiently values the reality of mental disorders^(6–8)^.

Also associated with the factor, aspects such as inadequate treatment, omissive professional behavior^(22)^ and other situations of social vulnerability of users represent generators of moral distress in nurses working in *RAPS* ^(9,22)^. These situations contribute to the low adherence of users to the services, leading to therapeutic failures that gradually progress to more serious mental disorders and require complex interventions such as hospital admissions, thus generating a cycle of new conflicts^(24,25)^.

In this context, it is worth noting that the potential risk of aggression to which nurses are exposed while providing assistance to users with violent behavior represents stressful experiences for their health, particularly when they lack management abilities to handle such a situation^(15,26)^. This fact is corroborated by the predictor “Experience situations of physical and/or verbal aggression by the user/family member in relation to professionals”, which presented a moderate median for moral distress in this study.

In mental health, the user’s and team’s safety is weakened when technical training is inadequate to address psychiatric conditions^(17,26)^. Therefore, “Professional ethical competence” is related to the global context of safety, considering that the team’s knowledge is significant to minimize events arising from practice. In this context, studies show that mental health nurses who are insecure in their work are more likely to develop moral distress^(12,17,26)^. In the present study, the predictors of this factor showed moderate values of moral distress, associating the block with the negative experience of nurses when working with professionals with no profile or unprepared to meet the demands.

The impact of these situations on nurses is closely linked to what each situation represents in their personal and professional condition and how each professional handles and uses their resources. Thus, for a less stressful work environment to be structured, permanent health education strategies that address ethical education, the problematizing teaching, and spaces for reflection on practice are required. Consequently, the knowledge emerging from this process will contribute to refining ethical and professional skills, encouraging nurses to explore morally correct actions^(24,26)^.

The factor “Conflicts with management” had the second lowest median for moral distress among the factors, with a significant association between the variables age and time working in the main job. In *RAPS* services, organizational and relational aspects have a direct impact on the quality of care provided by operational centers to users and their families. In this conception, conflicting relationships that go beyond the limits of ethical relationships, the fragility of interpersonal relationships, disrespect, lack of professional autonomy, and institutional practices that make qualified care unfeasible contribute to an environment that generates suffering. At this point, the significant role managers play in providing resources and building an institutional ethical climate is highlighted^(15)^, which promotes professional autonomy through ongoing health education processes and the implementation of assistive technologies^(16)^. Therefore, it should be noted that if management is dynamic and efficient, the nurses’ work process in RAPS tends to be more organized, promoting greater autonomy and safety for decision-making processes, thus minimizing situations that generate moral distress^(17)^.

Regarding the sample, it is worth mentioning that the participants in this study were mostly female (79.2%), aged between 30 and 39 years (52.6%), with nursing experience between 6 and 10 years (26.6%), similar to studies of moral distress in mental health nurses carried out in countries such as Japan^(10)^, South Korea^(11)^, Jordan^(12)^, U.S.A^(13)^, Norway^(15)^, and Thailand^(16)^. An Italian study of moral distress in mental health nurses was different from the majority, with a higher proportion of male participants (53.2%), aged between 41 and 50 years (51.4%)^(14)^.

This study also added a comparison analysis by groups, in which an association was identified between moral distress and age, number of employment relationships, type of employment relationship, length of service, weekly workload, and type of service. In general, studies on the subject did not identify an association between the variables sex, age, professional experience, and predictors of moral distress in nurses^(10,12–14)^. However, a Jordanian study found that the additional training was positively associated with moral distress and that younger nurses have greater moral distress^(12)^.

Regarding the level of moral distress in mental health nurses, a South Korean study^(11)^ showed average values considered moderate (3.74). In Japan, these professionals showed relatively low levels of moral distress, although with relative frequency, mostly associated with the predictor “Inadequate number of nurses for the demands of the service”^(10)^. In Jordan, the level of moral distress among mental health nurses was moderately high^(12)^. In contrast, in the United States^(13)^ and Italy^(14)^, these professionals had low and moderate levels of moral distress.

In view of the results presented by this and other studies on moral distress in mental health nurses, it is significant to raise the level of discussions about coping strategies for moral distress, seeking to create a protection network for these professionals. This way, moral sensitivity represents the first protective factor for nurses, equipping them to refine the perception and recognition of the ethical/moral problem, considering the most prudent choice for each case^(27)^. Even in the face of evidence that more sensitive nurses suffer more, moral sensitivity remains an advantage for professionals, as it activates cognitive structures, preparing professionals for decisions that minimize the negative effects of moral distress^(23,28)^.

In the context of coping with moral distress, moral resilience is presented as the ability to manage stressors arising from practice, so that moral courage is developed. Conceptually, moral resilience seeks to respond to ethical/moral problems to preserve the professional’s integrity and avoid suffering^(29)^.

Moral courage, in its turn, involves the nurse’s ability to face ethical/moral problems, overcome fear, endure suffering, stand up for their values, and be morally prudent in situations that would lead them to act otherwise. The effect of moral courage includes the moral commitment, assertive decision-making, the patient’s, user’s, and professional’s comfort and safety^(30)^.

In view of the above, it is worth noting that more sensitive, resilient, and morally courageous nurses are able to equip themselves for ethical decision-making in health and deliberate on facts, with greater discretion and confidence, thus reducing moral distress.

As a limitation of the study, its originality in the field of mental health in Brazil is believed to have made comparative analyses in the national scenario impossible. Therefore, further investigations on moral distress in nurses in this work context are suggested, considering the dissimilar distribution of *RAPS* in Brazilian regions with different socioeconomic and cultural characteristics, and needs. Thus, it is believed that the object’s coping strategies shall undergo in-depth discussion. The implication of the study in practice gives way to reflection on the performance of mental health nurses in Brazil, in addition to encouraging the construction of policies aimed at qualifying all dimensions of care, thus reducing the undesirable effects of moral distress.

## CONCLUSION

The present study allowed the analysis of the frequency and intensity of moral distress in nurses who work in different mental health services in Brazil, focusing on the most severe predictors of the phenomenon. It was found that these professionals have mostly moderate levels of general moral distress in their daily practice.

The factors “Working Conditions” and “Social Conflicts” are associated with the predictors with the highest median of moral distress among the studied sample. In this sense, it is worth pointing out that the complex and specific work spaces of *RAPS* need an ethical, technical, and humanized environment, where institutional impediments are rethought to transform the work process and nurses can act according to their moral values. In this wise, ethical problems emerging from care practice, when perceived by more prepared and confident nurses, tend to be prudently deliberated.

In the opposite direction, the predictors with lower levels of moral distress among the studied sample were associated with the factor “Defense of values and rights”, indicating that the nursing practice in these spaces, historically directed to its improvement, has sought to guarantee and respect the global rights of users, even though there are situations that cause moral discomfort.

The evidence that moral distress reaches and affects, in different proportions, Brazilian nurses working in mental health services demonstrate the dimension and plurality of the problem. Therefore, the relevance of discussions and reflections on coping strategies for moral distress is highlighted, articulating elements such as sensitivity, resilience, and moral courage, so that the ethical health decision-making process becomes more effective in care and management settings.
